# Production of new antimicrobial palm oil-derived sophorolipids by the yeast *Starmerella riodocensis* sp. nov. against *Candida albicans* hyphal and biofilm formation

**DOI:** 10.1186/s12934-022-01852-y

**Published:** 2022-08-17

**Authors:** Achmad Rifky Alfian, Kwanrutai Watchaputi, Chayaphathra Sooklim, Nitnipa Soontorngun

**Affiliations:** Gene Technology Laboratory, Biochemical Technology Division, School of Bioresources and Technology, King Mongkut’s University of Technology Thonburi, 49, Tian Talay Road, Tha Kham, Bang Khuntian, Bangkok, 10150 Thailand

**Keywords:** Antifungal, Biosurfactant, *Starmerella riodocensis*, *Starmerella bombicola*, Sophorolipid production, Yeast cell factory

## Abstract

**Background:**

Microbial derived-surfactants display low eco-toxicity, diverse functionality, high biodegradability, high specificity, and stability under extreme conditions. Sophorolipids are emerging as key biosurfactants of yeast origins, used in various industrial sectors to lower surface tension. Recently, sophorolipid complexes have been applied in biomedicals and agriculture to eradicate infectious problems related to human and plant fungal pathogens. This study aimed to characterize the functional properties and antifungal activities of sophorolipids produced by a newly characterized *Starmerella riodocensis* GT-SL1R sp. nov. strain.

**Results:**

*Starmerella riodocensis* GT-SL1R sp. nov. strain was belonged to *Starmerella* clade with 93.12% sequence similarity using the ITS technique for strain identification. Sophorolipids production was examined, using co-carbon substrates glucose and palm oil, with a yield on the substrate between 30 and 46%. Using shake-flasks, the *S. riodocensis* GT-SL1R strain produced biosurfactants with an emulsification activity of 54.59% against kerosene compared to the *S. bombicola* BCC5426 strain with an activity of 60.22%. Maximum productivities of GT-SL1R and the major sophorolipid-producer *S. bombicola* were similar at 0.8 gl^−1^ h^−1^. *S. riodocensis* GT-SL1R produced mixed forms of lactonic and acidic sophorolipids, shown by TCL, FTIR, and HPLC. Importantly, the complex sophorolipid mixture displayed antifungal activity against an opportunistic yeast pathogen *Candida albicans* by effectively reducing hyphal and biofilm formation.

**Conclusions:**

Sophorolipids derived from *S. riodocensis* demonstrate potential industrial and biomedical applications as green surfactant and antifungal agent. Since numerous renewable bioresources and industrial wastes could be used by microbial cell factories in the biosynthesis of biosurfactants to reduce the production cost, sophorolipids hold a promising alternative to current antimicrobials in treatments against infectious diseases in humans, animals, and plants.

## Introduction

Biosurfactants could be synthesized by microorganisms, including bacteria, yeast, and filamentous fungi. They are widely known as surface-active amphipathic compounds that function as micelles in a heterogeneous system and act to reduce the interfacial and surface tension [1]. Biosurfactants consist of different chemical structures in various forms of fatty acids, glycolipids, lipopeptides, neutral lipids, polysaccharide-protein complexes, polymeric surfactants, and phospholipids [2]. These compounds also have better advantages over chemical surfactants due to their environment-friendly features and versatile functions. Biosurfactants display low toxicity, diverse functionality, high biodegradability, stability, and specificity. They can be synthesized from numerous renewable sources as well [1, 3]; thereby providing many possibilities for future applications.

Biosurfactants have been applied in various industrial sectors according to their chemical structures and functional properties, including in biomedical-related areas. They possess promising biological functions against pathogenic microbes, such as anti-adhesive, anti-biofilm, and anti-microbial activities [4, 5]. Sophorolipids (SLs) are promising biosurfactant classes that belong to the glycolipid family and are produced mainly by yeasts. Various yeast species have been reported to produce SLs, including *Starmerella apicola*, *Pseudohyphozyma bogoriensis*, *S. riodocensis, S. stellata*, *S. floricola*, and *S. batistae* [2, 6, 7]. *S. bombicola* (formerly known first as *Torulopsis bombicola* and then as *Candida bombicola*) has been extensively studied due to its ability to produce high amounts of SLs with excellent surface lowering properties and an environmental-friendly profile [8, 9]. SLs comprise a dimer of sophorose, a disaccharide with two glucose units linked by the β-1,2 bond and a long-chain fatty acid. In fact, SLs have different formations depending on the presence or absence of lactonization, the degree of acetylation of the disaccharide, fatty acid chain length, saturation, and the position of the fatty acid hydroxyl group (ω or ω-1) [8]. Moreover, the carboxylic end of fatty acids can be formed at different positions such as the esterified 4″ position of the lactonic ring structure. Alternatively, the carboxylic form can be in a free form as the acidic structure [1, 10].

To date, the petroleum industry remains the largest market for biosurfactants. It is mainly due to their wide array of applications in bioremediation and dispersion of oil spills as well as removal and mobilization of oil residues in storage tanks and oil recovery. Biosurfactant applications in other industries like food, cosmetics, medical, and pharmaceuticals, gradually apply these biomolecules. One of the most prominent biosurfactants, namely SLs, has broad antimicrobial activity against pathogenic fungi, bacteria, and parasites [1, 11–17]. Basically, SLs derived from *S. bombicola* ATCC 22,214 have different antimicrobial actions depending on the SL types. Lydon et al. [14] reported the potential of acidic SLs as antimicrobial agent. Non-acetylated acidic C_18:1_ has antimicrobial activities against the nosocomial infective agents *Pseudomonas aeruginosa* and *Enterococcus faecalis*. Lactonic SL, on the other hand, effectively inhibited cell growth of *Bacillus subtilis* and *Propionibacterium acne* compared to acidic SL [15]. Additionally, Tang et al. [16] reported that modified SLs (ester and acid forms) possessed good antibacterial activity against the Gram-positive bacterium, *B. subtilis*. Meanwhile, lactonic SL-ester displayed excellent antibacterial activity against the plant disease bacterium *Moesziomyces* sp. than the acidic SLs.

SLs have been applied as alternative compounds to overcome the infectious problems related to *Candida albicans* and some pathogenic yeasts [18]. It is reported that they can inhibit *C. albicans* biofilm formation and reduce the viability of preformed biofilms. Besides, when SLs are combined with clinical antifungal drugs, such as amphotericin B (AMB) or fluconazole (FLZ), they effectively inhibit biofilm formation and preformed biofilms of *C. albicans*. Moreover, SLs also downregulate the expression of several *C. albicans* hypha-specific genes [18]. *C. albicans* is a polymorphic organism that adheres to different surfaces including in the human body and medical devices like catheters, cardiac pacemakers, endotracheal tubes, central nervous system, and heart valves [19]. *C. albicans* is one of the most known species which causes candidiasis, a fungal infection and current medical challenge. The spectrum of disease caused by invasive candidiasis is estimated between 40% to 70% from minimally symptomatic candidemia to sepsis-associated mortality [20, 21]. The stages of *C. albicans* forms of yeast, hyphae, and pseudohyphae can be distinguished from their cell morphology, function, and growth conditions. During *C. albicans* life cycle, hyphae is a critical phase in the infection process that can cause tissue damage by invading mucosal epithelial cells and subsequently leading to blood-stream infection [21]. Beside *C. albicans*, the SLs have been recently reported to exhibit promising antifungal activity against plant and human pathogenic fungi such as *Colletotrichum gloeosporioides, Fusarium verticilliodes, Fusarium  oxysporum, Fusarium  solani, Corynespora cassicola,* and *Trichophyton rubrum* [1, 22]. Furthermore, the anti-dermatophyte activity of SLs biosurfactant of *Rhodotorula babjevae* YS3 is used in the antifungal treatment against cutaneous mycoses caused by *Trichophyton mentagrophytes* [17]. Moreover, de O Caretta et al. [11] report that the antimicrobial activity of SLs against phytopathogens of cherry tomato, including *Sclerotium rolfsii*, *Botrytis cinerea*, *Rhizoctonia solani* and *Pythium ultimum*.

Van Bogaert et al. [9] reported that when both hydrophobic vegetable oil and hydrophilic glucose are used as substrates, the production yields of SLs are generated over 400 g l^−1^, using *S. bombicola*. In the present study, we have investigated the use of mixed glucose and palm oil as a low-cost renewable substrate that acts as a carbon source in the bioconversion process of SLs due to the abundance of palm products and wastes. We first examined the physico-chemical characteristics of SLs as a biosurfactant produced by the newly isolated *S. riodocensis* strain GT-SL1R*,* based on oil displacement and emulsification index. Then, antimicrobial properties of SLs against the opportunistic fungi *C. albicans* were investigated, including hyphal and biofilm formation. Colorimetric XTT reduction and crystal violet (CV) assays were used for quantifying the biofilm growth and eradication measurement of biofilm’s structure, respectively. Finally, scanning electron microscopic (SEM) assays were performed to visualize the cellular and biofilm morphology of *C. albicans*.

## Methods

### Microorganisms

*S. bombicola* yeast was purchased from the Thailand Network on Culture Collections (TNCC) cat no. BCC 5426. The yeast strain *S. riodocensis* strain GT-SL1R was obtained from the collection of Gene Technology Laboratory, KMUTT. *C. albicans* ATCC 90,028 was used in this study to test the antimicrobial activity of SLs. All strains were maintained in 25% (v/v) glycerol and kept at −20 ℃. The strains were regularly cultured on a YPD agar slant containing 10 g l^−1^ yeast extract, 20 g l^−1^peptone, 20 g l^−1^glucose, and 20 g l^−1^agar.

### Preparation of the inoculum and biosurfactant production

Preparation of the inoculum and biosurfactant production was carried out according to Shah et al. [23]. The inoculum medium contained (in g l^−1^) glucose 100, yeast extract 10, and urea 1. The culture was incubated at 30 °C for 24 h in a rotary shaker at 150 rpm. Afterward, cells were measured by dilution to an OD_600_ of 0.1 (1 × 10^6^ cells ml^−1^) and were transferred to a flask with 50 ml production medium. The production medium of 50 ml consisted of palm oil 100 (g l^−1^), glucose 100 (g l^−1^), yeast extract 10 (g l^−1^), and urea 1 (g l^−1^). Biosurfactant production was carried out at 30 °C in a rotary shaker at 150 rpm.

### Sophorolipid purification

Purification of the crude mixture of SLs biosurfactant was carried out by silica gel column chromatography (200–300 mesh size) as previously reported by Shah et al. [23]. Before loading the crude, the column was run by the eluent (chloroform). After that, 1 ml of the crude mixture SLs was dissolved in 1 ml of ethyl acetate and then loaded into the column. SLs were eluted in chloroform and methanol and carried out using a gradient system (0–20% methanol). The monitoring of different components of the crude mixture of SLs obtained from the column was carried out using thin layer chromatography (TLC) on Merck silica gel plate 60 F254. The mobile phase solvents used were chloroform, methanol, and water at the following ratio: 65, 15, 2, respectively. Silica gel was then sprayed with *p*-anisaldehyde for the detection of sugar moieties and heated for 5 to 10 min at 110 °C. The lipid fractions on the silica were exposed to iodine vapor.

### Determination of the yeast cell dry weight and SLs extraction

For biomass determination, 50 ml of cell culture was centrifuged at 6000×*g* for 15 min. The pellet was treated by washing twice with distilled water and then dried in an oven at 80 ℃ for 24 h. The supernatant was collected for SLs extraction. The dried cells were weighed using an analytical balance.

For SLs extraction, the supernatant was extracted with a comparable volume of ethyl acetate twice using a separating flask. The ethyl acetate phase collection was then vacuum dried at 40 °C to remove the solvent completely. After vacuum evaporation, the residual obtained during extraction was washed with hexane and after that removing the hexane was carried out by vacuum-drying at 40 °C. The SLs yields and biomass (g l^−1^) were obtained [24].

### Characterization of SLs

The presence of different functional groups in the crude mixture of SLs was determined by Fourier Transform Infrared (FTIR) spectroscopy (PerkinElmer). The range of FTIR spectra was performed in the 400–4000 cm^−1^ with attenuated total reflectance (ATR) mode. Subsequently, a crude mixture of SLs and a purified form of SLs extracted from the crude mixture produced by *S. riodocensis* were characterized by HPLC (Shimadzu, Japan) with a UV detector (207 nm) and a UPS C18 column (VertiSep ™, 5 μ, 4.6 × 250 mm) using gradient elution. The mobile phase was a linear gradient of 20% acetonitrile in H_2_O for 15 min, followed by 20–80% acetonitrile for 25 min, and then 80–100% acetonitrile for 10 min. The final mobile phase was 100% acetonitrile for 20 min at a flow rate of 0.5 ml/min [25].

### Oil displacement assay for biosurfactant screening

The strain was evaluated for its ability to produce biosurfactants using a simple oil displacement assay [2]. This assay was to evaluate the ability of the yeast strain to displace palm oil in a Petri dish with a diameter of 100 mm. Shortly, 20 ml of distilled water was added to Petri dishes, involving 20 µl of palm oil. Next, 10 µl of cell-free supernatants from cultures containing the positive treatment (palm oil) and without palm oil were separately added to the surface of distilled water. The results were considered positive for biosurfactant production by the appearance of a clear zone surrounded by palm oil.

### Emulsification index (E_24%_) measurement

To assess the emulsification activity of the culture, 2 ml of cell-free supernatant was added to the same amount of a kerosene hydrocarbon in a test tube. The mixed liquid was carried out by vortex for 2 min, followed by left undisturbed for 24 h. E_24%_ was determined as the stability of the emulsion layer by calculating its height divided by the total mixture and then multiplied by 100. This assay was completed in duplicates [26].

### Fatty acid composition

The fatty acid composition of oil and its derived extracted crude SLs was determined by gas chromatography equipped with a flame ionization detector (GC–FID, Shimadzu Inc., Tokyo, Japan). The fatty acid methyl ester of palm oil and SLs was prepared by esterification; the palm oil or sophorolipid [30 mg] were dissolved in 3 ml of toluene. The solution was added with 2% sulfuric acid which was prepared in methanol (1 ml) and the solution was reacted in a 70 ℃ water bath shaker for 4 h. After the reaction, wash the toluene phase with 3 ml of DI water thrice and discarded the water phase, and added NaSO_4_. The extracts were analysed by a GC–FID and the operating conditions of the GC were as follows: column was BPX70 (70% cyanopropyl polysilphenylene-siloxane; 30 m × 0.25 mm inner diameter × 0.25 μm film thickness). The column temperature was 160 ℃ (2 min), 2 ℃/min to 210 ℃; carrier gas was He at a flow rate of 1.04 ml/min.

### Anti-hyphal activity of SLs

RPMI-1640 medium with L-glutamine without sodium bicarbonate (Sigma) was prepared in DI water (15–20 ℃) and 34.53 gr of MOPS (Sigma) was added. The final pH was adjusted with NaOH to a pH of 7 and added additional water to 1 l of the final volume solution. A stock solution of extracted SLs produced by *S. riodocensis* in dimethyl sulfoxide (DMSO, Sigma) was prepared and kept at −20 °C until use. Hyphal growth assay was formed in 5 ml of RPMI-1640 medium and RPMI-1640 medium supplemented with 10% fetal bovine serum (FBS) [18]. Cell suspension was diluted at 1 × 10^6^ cells ml^−1^ in medium and incubated with different concentrations of purified SLs (0 μg ml^−1^, 8 μg ml^−1^, 16 μg ml^−1^, 32 μg ml^−1^, 62.5 μg ml^−1^, 125 μg ml^−1^, 250 μg ml^−1^, 500 μg ml^−1^, and 1000 μg ml^−1^) at 37 °C with agitation at 200 rpm for 5 h. Aliquots of samples were visualized under a bright-field using a 40X objective lens by Huma Scope Premium microscope and photographed.

### The antifungal activity of SLs against *Candida albicans* biofilm

Just prior 96-well assay, cells were grown overnight in RPMI-1640 media supplemented with 2% glucose at 37 ℃ according to previous work [27, 28]. The biofilms were formed in 96-well microtiter plates. Biofilm growth of *C. albicans* was quantified with an XTT-reduction assay [29] and the measurement of biofilm biomass was assessed with a crystal violet (CV) assay [30]. The cell suspension was prepared in RPMI-1640 medium at a cell density of 1 × 10^6^ cells ml^−1^. The XTT assay was used for determining the antifungal activity of SLs against *C. albicans* biofilm. 100 µl of cell suspension was distributed onto microtiter plate wells and incubated for 90 min (adherence phase) and 24 h at 37 °C. At the end of incubation, the plate was gently flicked to remove the medium, and biofilm was washed 3-times with sterile PBS to remove the nonadherent cells. Residual PBS of the wells was dried for 45 min at the end of the washing steps. Further, 100 µl of serially double-diluted concentrations of SLs were added to the wells of biofilms. In the chosen wells of biofilms, 100 µl of RPMI-1640 media containing final 5% DMSO without SLs was used as a control. Microtiter plates were incubated for 24 h at 37 °C. Colorimetric XTT [2,3-bis(2-methoxy-4-nitro-5-sulfophenyl)-2H-tetrazolium- 5-carboxanilide sodium salt] reduction assay was used to quantify the metabolic activity of biofilms.

As previously described [18] with slight modification, a colorimetric XTT reduction assay of biofilm was carried out as follows: a 0.5 g ml^−1^ stock solution of XTT tetrazolium salt was prepared in 1X PBS and filter sterilized using a 0.22 m pore size filter. Keep the stock solution in aliquots at −20 °C. Subsequently, an aliquot was thawed and a 1 µM final concentration of freshly prepared menadione was added to the XTT working solution before the experiment. 100 µl of the XTT/menadione solution was added to each well containing a prewashed biofilm as well as to the negative control (blank), using a multichannel pipette. The plates were covered with aluminium foil and incubated at 37 °C for 2 h in the dark. Further, 75 µl of the coloured supernatant was removed from each well with a multichannel pipette and placed on a new plate. The values were read using a microtiter plate reader at the wavelength of 492 nm.

The quantification of biofilm biomass after SLs treatment was carried out according to Khan et al. [30]. The medium was discarded from the wells after the proper incubation of the microtiter plates. Nonadherent cells were eliminated by washing the biofilms 3-times with 200 µl phosphate buffer saline (PBS) before being air-dried for 45 min. Subsequently, 0.4% aqueous crystal violet was used for staining the wells (110 µl each well) for 45 min. After that, the samples were washed four times with sterile distilled water (300 µl) followed by destaining with 95% ethanol (300 µl) for 45 min. 100 µl of the destaining solution was transferred to a new well therefore the amount of crystal violet stain in the destaining solution was measured at 595 nm using a microtiter plate reader.

### Analysis of scanning electron microscopy (SEM)

Poly-L-lysine (PLL) coated glass cover slips were used for substrate to form biofilms in 6-well cell culture plates. Glass coverslips were prepared as described by Dong et al. [31]. Glass coverslips (22 × 22 mm) were cleaned in 1 M HCl for 1 min, then immersed in piranha solution (3:1 (V/V), H_2_SO_4_:H_2_O_2_) for 1 h. Coverslips were rinsed with deionized water, methanol, and acetone, followed by air-dried and autoclaved for 15 min. Glass coverslips were then immersed in 0.1% PLL solution for 10 min and air-dried overnight before being rinsed with sterile deionized water and air-dried again. Coated coverslips were sterilized by UV radiation for 1 h under laminar air flow and put into the well plates for performing the biofilms for 90 min and 24 h at 37 ℃. At the end of incubation, wells containing coverslips were washed 3 times with PBS. 2 ml of different concentrations of SLs (0 µg ml^−1^, 64 µg ml^−1^, 125 µg ml^−1^, and 500 µg ml^−1^) were dispersed into the wells. RPMI containing 5% of DMSO without SL was included as control. Plates were incubated for 24 h at 37 ℃ followed by washing the biofilms 3 times with PBS. PBS washed biofilms were fixed with 4% of formaldehyde overnight. Rinse coverslips twice with PBS and once with distilled water for 10 min/each. Subsequently, coverslips were dehydrated in a graded series of ethanol solutions (30%, 50%, 70%, 95%, and 100% for 10 min/each followed by 3 changes at 100%). Coverslips were dried with critical point dryer (Leica model EM CPD300, Austria). Thereafter, samples were mounted and coated with gold (sputter coater, Balzers model SCD 040, Germany) and were visualized by scanning electron microscope (JSM-IT500HR InTouchScope™).

### Data and statistical analysis

Quantification data were calculated as mean values with standard deviation from at least two independent experiments, performed in triplicates (Microsoft Excel 365). Data were calculated and compared with those of control groups. The student’s t-test was used for determining the emulsification index, SLs production and biofilm formation. The cell dry weight (CDW), emulsification index, and oil displacement test were analysed by one-way ANOVA. A statistically significant p-value of 0.05 was used.

## Results

### Novel yeast strain *Starmerella riodocensis* GT-2564R from honeybee sample

The dry weight of honey mainly consists of carbohydrates (95–98%) while secondary metabolite agents and minerals make up the remaining 2–5% [32]. Recently, many beneficial yeasts and bacteria are reported in honey and honeybee products [33, 34]. As noted formerly by Pimentel et al. [35], *S. riodocensis* species, belonging to the *Starmerella* clade, was present in pollen-nectar provisions, larvae, and fecal pellets of nests of *Megachile* sp. [35]. Our previous study has uncovered various strains of *Saccharomyce*s and non-*Saccharomyces* species from honeybee samples. Many of which produce valuable biochemicals and bioactive compounds including, antimicrobial acids [33]. In the present study, among many yeast isolates obtained from the *Apis dorsata* raw honey samples, the GT-SL1R strain was selected and further characterized for the production of SLs biosurfactant. Sequencing and analysis of ITS regions of the 18S (1609–1627) and 28S (287–266) rRNA previously shown by Kurtzman & Robnett [36] were performed to construct a phylogenetic tree based on genomes publicly available in NCBI. The ITS sequences of the GT-SL1R strain was compared to those of closely related species in the GenBank database using the BLASTn program. GenBank was used to find sequences of their closest relatives and other genetically important species. GT-SL1R strain was closely related to *S. riodocensis* with 93.12% sequence similarity and differ from it by 17 nt in the ITS region, indicating the possibility of new species.

**Fig. 1:**
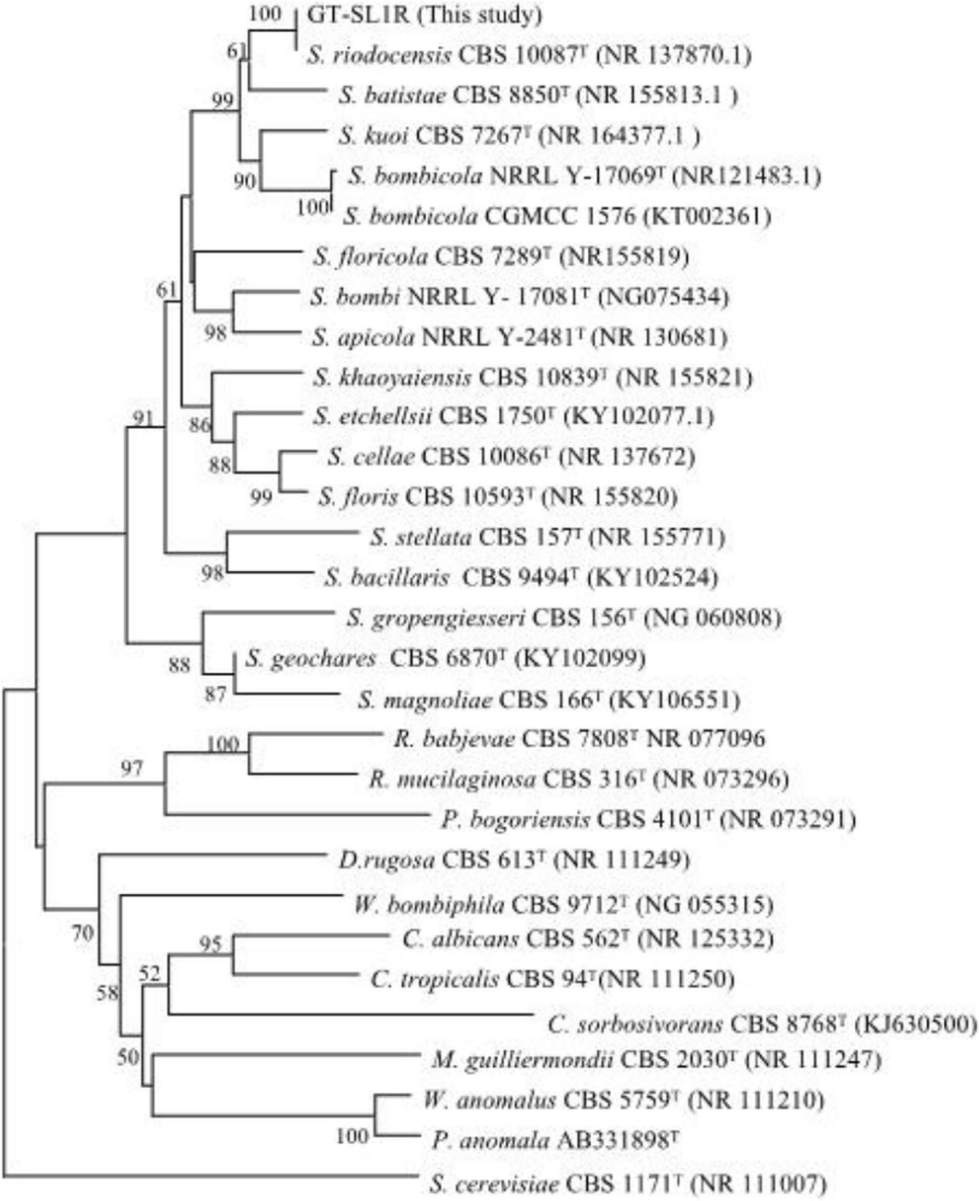
The phylogenetic tree displayed the relationships between the identified yeast isolate GT-SL1R strain of *S. riodocensis* and different yeast species. For comparison, based on ITS region sequences of type strains from different species which are retrieved from the literature and NCBI database. The phylogenetic tree was constructed using the neighbor-joining method. Bootstrap values were calculated from 1000 replicates. T symbol indicated “Type Strain” that is used as a reference

Although nucleotide sequences of ITS are usually sufficiently conservative to identify related species, a particular region is important for phylogenetic analysis. In our work, the phylogenetic analysis presents all 28 species (Fig. 1) and was separated into two subclasses, one showed *Stamerella* sp. clade including the GT-SL1R strain, and the other presented member of sophorolipids yeast producers [6, 37]. Indeed, many members of both clades were potential biosurfactant producers with useful applications in the cosmetic, pharmaceutical, food, and cleaning industries [3]. As shown, Clade 1 *S. bombicola* clade includes *S. bombicola* NRRL Y-17069^T^ (NR121483) and all the former type trains of *Starmerella* species including *S. bombi* NRRL Y- 17081^T^ (NG075434) [35], *S. apicola* NRRL Y-2481^T^ (NR130681) [38], *S. khaoyaiensis* CBS 10839^T^ (NR155821) [39], *S. cellae CBS* 10086^T^ (NR137672) [35], *S. floris* CBS 10593^T^ (NR155820) [39], *S. batistae* CBS 8850^T^ (NR 155,813.1), *S. stellata* CBS 157^T^ (NR 155,771), *S. kuoi* CBS 7267^T^ (NR 164,377.1), *S. floricola* CBS 7289^T^ (NR155819), *S. etchellsii* CBS 1750^T^ (KY102077.1), *S. bacillaris* CBS 9494^T^ (KY102524), *S. gropengiesseri* CBS 156^T^ (NG 060,808), *S. geochares* CBS 6870^T^ (KY102099), *S. magnoliae* CBS 166^T^ (KY106551) [6, 37] (Fig. 1). The phylogenetic relationship among these yeast strains in 8 species presented here were suggested to possess potential metabolic activity as great biosurfactant producers. Namely, *S. batistae* CBS 8550^T^ (NR155813) [39], *P. bogoriensis* CBS 4101^T^ (NR 073,291) [40], *S. apicola* NRRL Y-2481^T^ (NR 130681) [35], *S. stellata* CBS 157^T^ (NR155771) [39], *C. tropicalis* CBS 94T(NR 111250), *Meyerozyma guilliermondii* CBS 2030T (NR 111247), *Wickerhamomyces anomalus* CBS 5759^T^ (NR111210) [41], *S. cerevisiae* CBS 1171 T (NR 111007) [42] were among the widest research yeasts for biosurfactant biosynthesis [3] in addition to *S. bombicola* and *S. riodocensis*.

### SLs biosurfactant produced by *Starmerella riodocensis* GT-SL1R

**Fig. 2:**
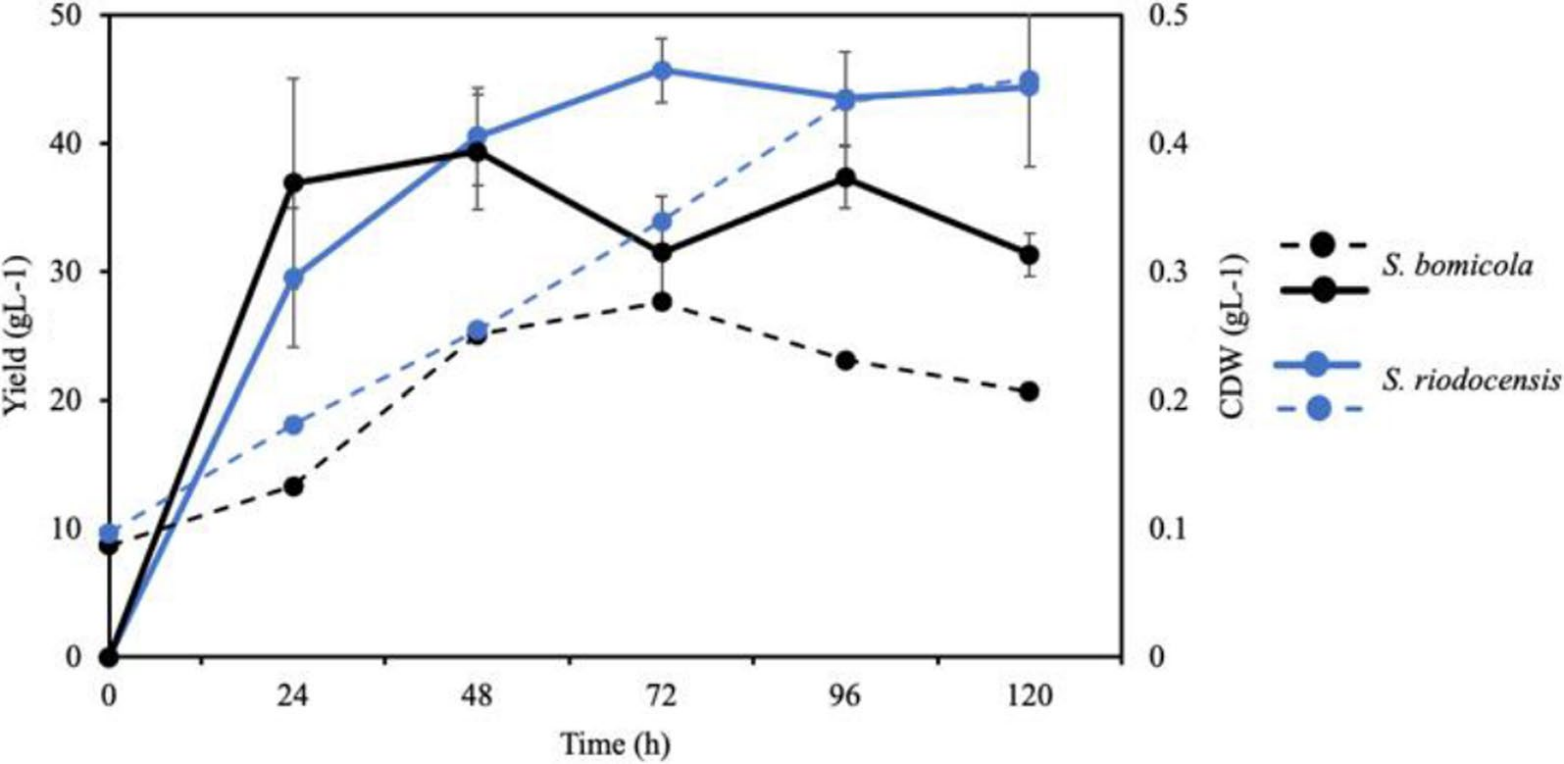
Biomass (dash line) and biosurfactant production (solid line) of *S. riodocensis* GT-SL1R strain (blue line) and *S. bombicola* BCC5426 strain (black line) grown at 30 °C, 150 rpm from 0 to 120 h. Cell growth was supplemented with palm oil as a hydrophobic substrate. The data were given as mean values obtained from at least two independent experiments performed in triplicates. Error bars displayed standard deviations of means

Previously, glucose and palm oil have been effectively used as hydrophilic and hydrophobic substrates, respectively, in SLs fermentation as reported by Shah et al. [23]. The *S. riodocensis* GT-S1R was then used to investigate the production of SLs biosurfactant using palm oil as a hydrophobic substrate. The yeast *S. riodocensis* appeared to grow exponentially until day 5 of cultivation supplemented with palm oil while the growth of *S. bombicola* remained stable and slightly dropped after day 3. The maximal cell biomass value was found at 0.45 g l^−1^ in *S. riodocensis* on day 5 (Fig. 2). When cultivated without palm oil, *S. riodocensis* showed a lower biomass accumulation of 0.25 g l^−1^ (data were not shown). After depletion of oil as a carbon source, the cell biomass dropped gradually, thus, supplementation of palm oil was necessary to maintain good growth and biomass of yeast cells. Next, the biosurfactant production by *S. riodocensis* GT-SL1R strain during fermentation using palm oil and glucose as the hydrophobic and hydrophilic substrates, respectively, was examined. The highest biosurfactant yield of 45.70 g l^−1^ produced by *S. riodocensis* was obtained on day 3 of cultivation, corresponding to the log phase of yeast growth (Fig. 2). Similarly, the highest crude mixture of biosurfactant yield of 42.81 g l^−1^ was produced by *S. bombicola* on day 2 of cultivation and then the production was decreased afterward (Fig. 2). A summary of SLs production was provided (Table 1).

**Table 1:** A list summarizing biosurfactant characterization and production of SLs

Parameter	Strain	
	*S. riodocensis* GT-SL1R	*S. bombicola* BCC5426
Emulsification index (%)		
24 h	32.50 ± 4.43*	48.48 ± 0.01
48 h	40.62 ± 1.80	52.58 ± 6.96
72 h	41.27 ± 7.68	55.88 ± 1.74
96 h	54.68 ± 3.12	55.88 ± 2.94
120 h	54.59 ± 3.13	60.22 ± 2.48
Oil displacement (cm)		
24 h	0.80 ± 0.12	0.83 ± 0.04
96 h	1.08 ± 0.25	1.10 ± 0.16
SL production (g l^−1^)		
24 h	29.54 ± 5.40*	36.89 ± 8.16
48 h	40.55 ± 3.82*	39.36 ± 4.48
72 h	45.70 ± 2.48*	31.51 ± 4.33
96 h	43.54 ± 3.64*	37.34 ± 2.40
120 h	44.44 ± 6.22*	31.34 ± 1.68
Productivities (gl^−1^ h^−1^)		
24 h	1.23	1.53
48 h	0.84	0.82
72 h	0.63	0.43
96 h	0.45	0.38
120 h	0.37	0.26
Yield on substrate (%)		
24 h	29.54	36.89
48 h	40.55	39.36
72 h	45.70	31.51
96 h	43.54	37.34
120 h	44.44	31.34

*p value of less than 0.05 between *S. riodocensis* GT-SL1R and *S. bombicola* BCC5426

### Identification of SLs congeners

**Fig. 3:**
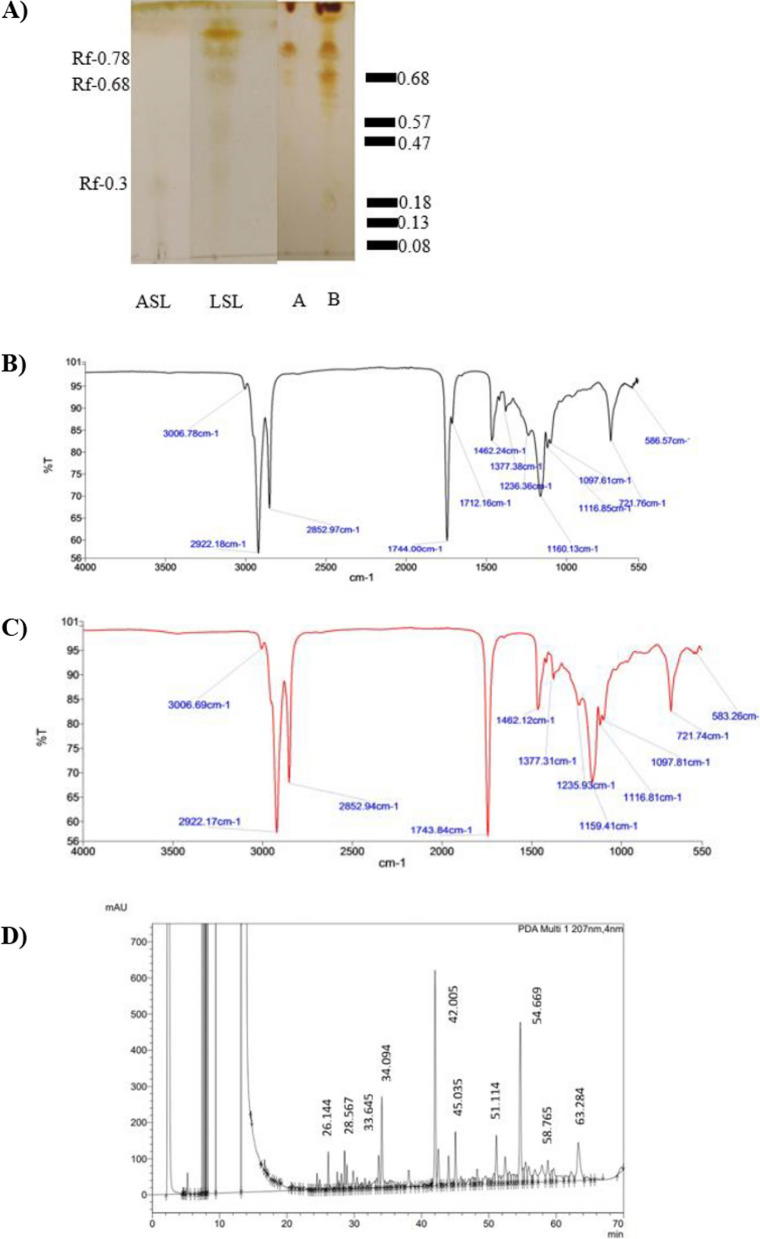
Structural characterization of SLs produced by *S. riodocensis* GT-SL1R strain using mixed glucose and palm oil. **A** TLC chromatograms showing the retention factors of SLs biosurfactants obtained from *S. bombicola* (**A**) and *S. riodocensis* (**B**) compared to acidic (ASL) and lactonic (LSL) SLs, respectively. Marker bands indicated previously identified congeners of SLs produced by *S. bombicola*. FTIR spectra of crude SLs produced by *S. riodocensis* (**B**) and *S. bombicola* (**C**). HPLC chromatogram of purified form SLs extracted from the crude mixture SLs produced by *S. riodocensis* GT-SL1R strain (**D**)

Thin layer chromatography (TLC) was preferably an initial characterization technique for the detection of biosurfactants before the structure elucidation of SLs using more advanced techniques. To examine the presence of SLs synthesized by *S. riodocensis* GT-SL1R strain, *p*-anisaldehyde reagents and iodine vapor were used for sugar and lipid detection, respectively [2]. As confirmed by the TLC chromatogram, the standard SLs (acidic SL non-acetylated and 1,4″-sophorolactone 6ʹ,6″-diacetate) were carried out to predict the chemical nature of the SLs biosurfactant [1]. The sample obtained by *S. riodocensis* GT-SL1R strain showed two spots of crude biosurfactant with retention factors (Rfs) of 0.68 and 0.78 (Fig. 3), comparable to Rfs of 0.49, 0.56, and 0.68 previously identified as lactonic SL by Sen et al. [1]. Moreover, an additional spot with an Rf of 0.18 was also identified from the crude sample produced by the *S. riodocensis* GT-SL1R strain (Fig. 3), corresponding to the Rf of 0.18 reported as lactonic SLs by Asmer et al. [43] or as acidic SL based on the standard.

For further investigation of key functional groups, FTIR spectroscopy of crude SLs produced by *S. riodocensis* GT-SL1R strain using palm oil as a hydrophobic substrate was obtained (Fig. 3B). The first peak at 3006.78 cm^−1 ^represented alkenes (= C-H stretch). The IR spectra of 2922.18 cm^−1^ and 2852.97 cm^−1^ corresponded to methylene groups (C-H stretch). Moreover, esters, lactones, or acids may correspond to the observed C = O stretch absorption band at 1744.00 cm^−1^. C-O–H in-plane bending of carboxylic acid (-COOH) was identified at 1462.24 cm^−1^. The C-O stretch absorption band can be found at 1236.36 cm^−1^ which may contribute to acetyl esters. The stretch at 1160.13 cm^−1^ was related to the C-O band of C(-O)-O-C in lactones. Furthermore, the C-O stretch of the C-O–H group of sugar could be observed at 1097.61 cm^−1^.

While FTIR spectroscopy of crude SLs produced by *S. bombicola* BCC5426 strain showed similar to FTIR spectra of crude SLs produced by *S. riodocensis* GT-SL1R strain (Fig. 3C). The first peak at 3006.69 cm^−1^represented alkenes (= C-H stretch). The IR spectra of 2922.17 cm^−1^ and 2852.94 cm^−1^ corresponded to methylene groups (C-H stretch). Moreover, esters, lactones, or acids may correspond to the C = O stretch absorption band at 1743.84 cm^−1^. C-O–H in-plane bending of carboxylic acid (-COOH) was identified at 1462.12 cm^−1^. The C-O stretch absorption band can be found at 1235.93 cm^−1^ contributing to acetyl esters. The stretch at 1159.41 cm^−1^ was related to the C-O band of C(-O)-O-C in lactones. Furthermore, the C-O stretch of the C-O-H group of sugar could be observed at 1097.81 cm^−1^. The structural details of both FTIR spectra of crude SLs were similar to the result in the previous report [44].

Subsequently, high performance liquid chromatography (HPLC) was performed to identify SLs purified from the extracted culture medium supplemented with palm oil. For HPLC analysis from palm oil-derived SLs produced by *S. riodocensis* (Fig. 3D) was compared with acidic (acidic SL non-acetylated) and lactonic (lactonic di-acetylated) standards (Carbosynth Ltd), respectively. Peaks were recorded at retention time (RT) at 26.144, 28.567, 33.645, 34.094, and 42.005 corresponding to acidic SLs, while 45.035, 51.114, 54.669, 58.765, and 63.284 corresponded to lactonic SL. From the recorded peaks, the RTs were not compatible with acidic non-acetylated and lactonic di-acetylated standards. We suggested that they were derivative of SLs. Kim et al. [25] also reported the HPLC analysis of SLs derived from rapeseed and waste-cooking oils and identified the area peaks of acidic and lactonic SLs at RT of 32.5–42.5 and 42.5–52, respectively.

Next, the result obtained from gas chromatography equipped with a flame ionization detector (GC–FID) showed that the palm oil contained 0.19% decanoic acid (C10), 0.25% lauric (C12), 0. 76% myristic acid (C14), 36.03% palmitic (C16), 0.14% palmitoleic (C16:1n7), 3.71% stearic (C18), 40. 91% oleic (C18:1n9), 1.61% vaccenic (C18:1n7), 15.04% linoleic (C18:2n6), 0.64% linolenic (C18:3n3), 0.32% arachidic (C20), and 0.14% eicosanoid (C20:1n9). SLs derived from palm oil have a similar fatty acid composition profile to palm oil, suggesting that SLs mainly contained palmitic and oleic acid derivatives.

### Emulsification index and oil displacement assay of SLs

Emulsification indices (E_24%_) were examined using cell-free supernatant during 5 days of cultivation against kerosene. As shown in Table 1, the emulsion index of biosurfactant produced by *S. riodocensis* GT-SL1R strain after 24-, 48-, 72-, 96-, and 120-h period of incubation was 13.8; 40.6; 41.2; 54.6; and 54.5%, respectively. The result indicated that the optimum production and activity of biosurfactant in terms of emulsion layer were achieved after 96 h of incubation and presented satisfactory or high activity (> 50%) as reported by Yalcin et al. [45] and Araujo et al. [26]. While the emulsion index of biosurfactant produced by *S. bombicola* after 24-, 48-, 72-, 96-, and 120- h periods of incubation increased with time from 48.4; 52.6; 55.8; 55.8; and 60.2%, respectively (Table 1). The result indicated that the optimum production and activity of SLs in terms of emulsion layer was achieved after 120 h of incubation which agreed with those presenting superior results (> 60%) as reported by Yalcin et al. [45].

Subsequently, the biosurfactant activity of *S. riodocensis* GT-SL1R and *S. bombicola* BCC5426 strains was tested by oil displacement assay (Table 1). The supernatant containing SLs of each yeast species was dropped on the top of oil at the centre of the petri dish. Both samples indicated a good ability to displace the oil from the surface while *S. bombicola* BCC5426 strain supernatant had better activity to displace the oil as compared to *S. riodocensis* GT-SL1R strain. Oil-free clearing zone diameters of *S. riodocensis* and *S. bombicola* strains increased with days of cultivation from 0.80 or 0.83 to 1.08 or 1.10 cm at day 4, respectively (Table 1).

### Antifungal SLs inhibited *Candida albicans* hyphal growth and biofilm formation

**Fig. 4:**
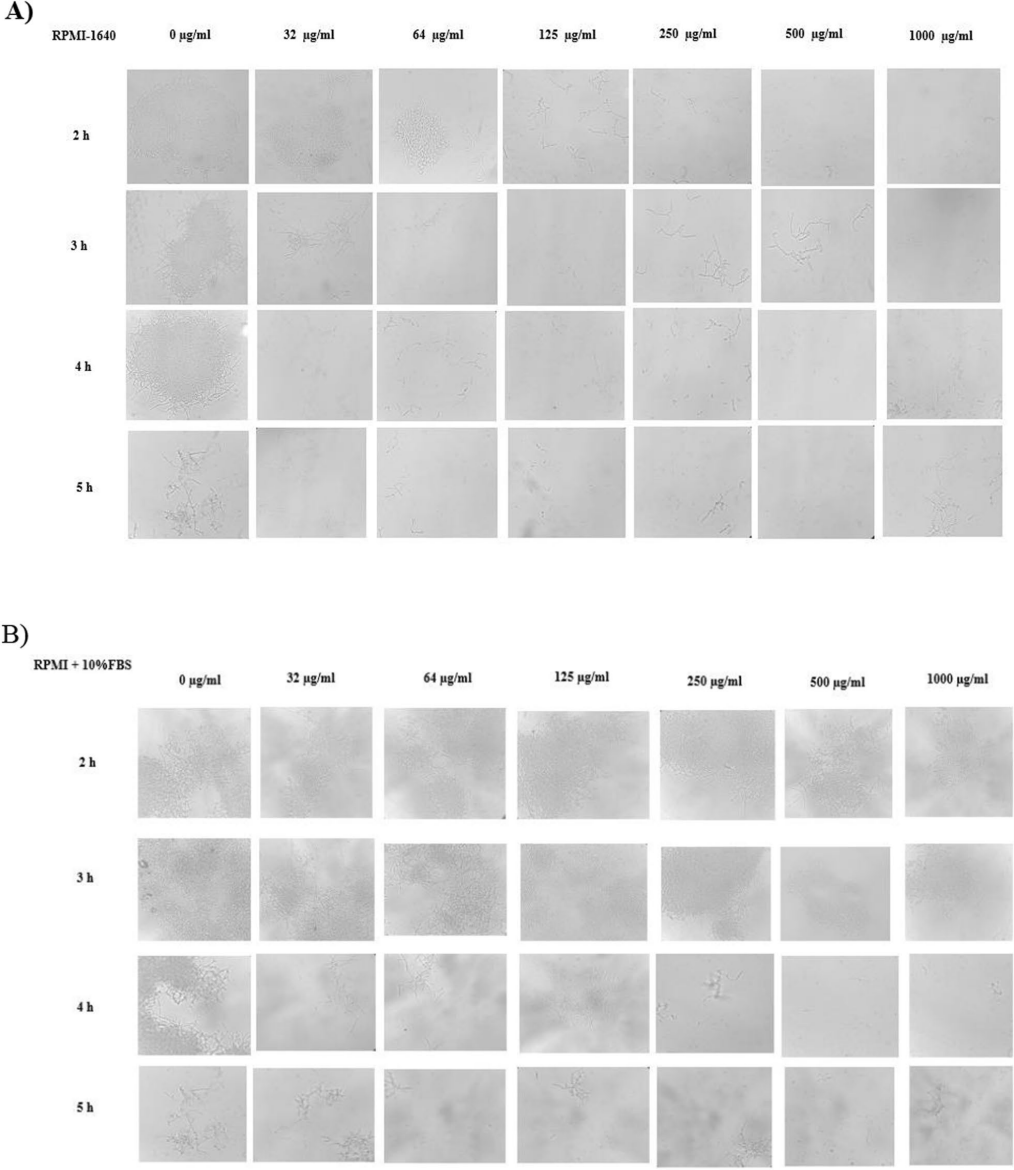
Effect of different concentrations of SLs produced by *S. riodocensis* GT-SL1R strain to inhibit *C. albicans* hyphal growth. *C. albicans* cells were grown in (**A**) RPMI-1640 media and (**B**) RPMI-1640 media supplemented with 10% FBS at 37 ℃ for a duration of 2-, 3, 4 or 5 h. Cells were observed using a microscope and photographed at 400×magnification. Representative images of two independent experiments performed in triplicates were shown

*C. albicans* is the main cause of superficial and life-threatening systemic infections. Since *C. albicans* biofilms are inherently resistant to most antifungals and this makes it difficult to treat. The effect of SLs produced by *C. riodocensis* GT-SL1R on hyphal growth inhibition was first investigated, followed by the inhibitory effect on biofilm formation. The effect of SLs on hyphal growth was examined using aliquots of the cells that are microscopically observed after 2, 3, 4, or 5 h of incubation by inducing with 10% FBS. The effect of SLs produced by *C. riodocensis* GT-SL1R on hyphal growth inhibition of *C. albicans* was increased with SLs concentration and incubation time (Fig. 4). Massive *C. albicans* hyphae were visible in control samples (0 μg ml^−1^ SLs), especially in the presence of 10% FBS was compared to the RPMI alone (Fig. 4). The increasing SLs concentrations diminished the hyphal growth, starting at a low concentration of 32 μg ml^−1^ of SLs, resulting in a lower appearance of the hyphal form of *C. albicans* cells. Subsequently, hyphal growth was largely inhibited at 500–1000 μg ml^−1^ of SLs treatment with reducing cell survival and remains in yeast form. Noticeably, upon 64 μg ml^−1^ of SL treatment, hyphae were shortened as compared to untreated (no SLs) and completely massively inhibited at higher SLs concentrations of 500 and 1000 μg ml^−1^ after 5 h of treatments (Fig. 4).

**Fig. 5:**
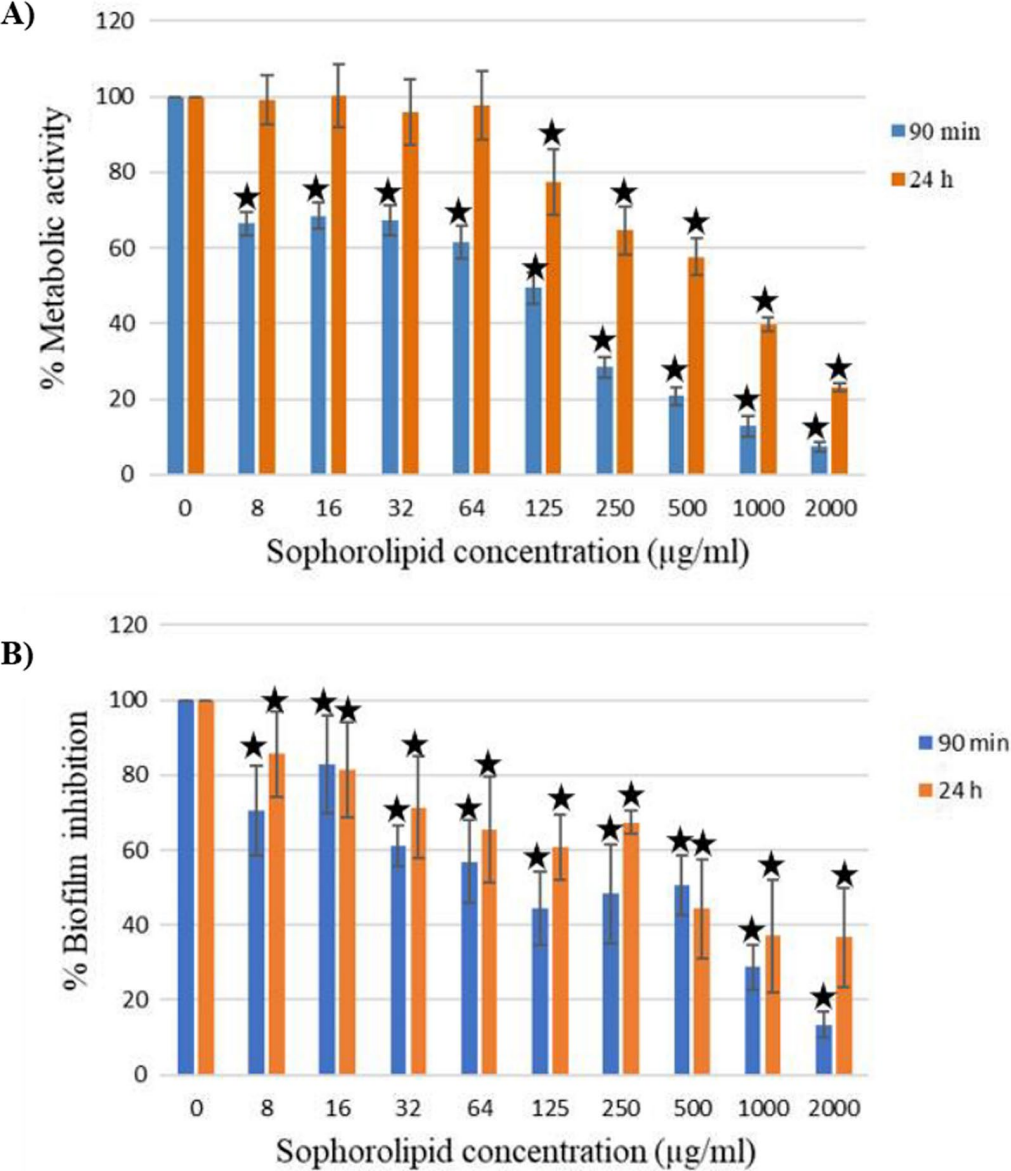
Antifungal activity of SLs produced by *S. riodocensis* GT-SL1R strain against *C. albicans* adherence phase (90 min) and mature biofilm at 24 h. **A** Readings of colorimetric XTT reduction assay at 492 nm are expressed in terms of % metabolic activity of control and (**B**) biofilm inhibition using crystal violet assay. Results represent the average of three independent experiments ± SD. *p < 0.05 when compared with SLs untreated controls

The effect of SLs produced by *S. riodocensis* GT-SL1R strain on *C. albicans* biofilm formation was then investigated. This test was to determine the inhibitory activity of SLs against *C. albicans* biofilm formation. In the presence of serially double diluted concentrations of SLs (0–2000 μg ml^−1^), biofilm formation was started in 96-well microtiter plates at 37 °C for 90 min (after the adherence phase) and 24 h. The metabolic activity of *C. albicans* was measured using a colorimetric XTT reduction assay that confirmed the inhibitory activity of SLs on the cell viability of *C. albicans*. It was found that 125 μg ml^−1^ of SLs can inhibit *C. albicans* 90-min-old biofilm by approximately 50% (Fig. 5A). Moreover, 500 μg ml^−1^ of SLs could reduce the viability of *C. albicans* in a mature biofilm by approximately 50% within 24 h as can be seen in Fig. 5. The adherence phase is the process of *C. albicans* to initiate biofilm formation when yeast cells adhere to a material surface [46]. The concentration of 500 μg ml^−1^ SLs could inhibit the mature biofilm after 24 h (Fig. 5A), when fully mature biofilms were formed, consisting of a thick network of yeasts, hyphae, and pseudohyphae. This phase is a critical step in the biofilm formation of *C. albicans*. Thus, SLs produced by *S. riodocensis* revealed a good potential to prevent the development of *C. albicans* biofilm. Further, the antibiofilm activity of SL was assessed using crystal violet. The capacity of crystal violet (CV) to stain the polysaccharide matrix makes it one of the most popular dyes to assess biofilm [47]. Our study revealed that 125 μg ml^−1^ of SLs can reduce *C. albicans* 90-min-old biofilm by approximately 50% while 500 μg ml^−1^ of SLs could reduce the biofilm of *C. albicans* in a mature biofilm by approximately 50% at 24 h (Fig. 5B).

### Effect of SL on biofilm formation and morphology

**Fig. 6:**
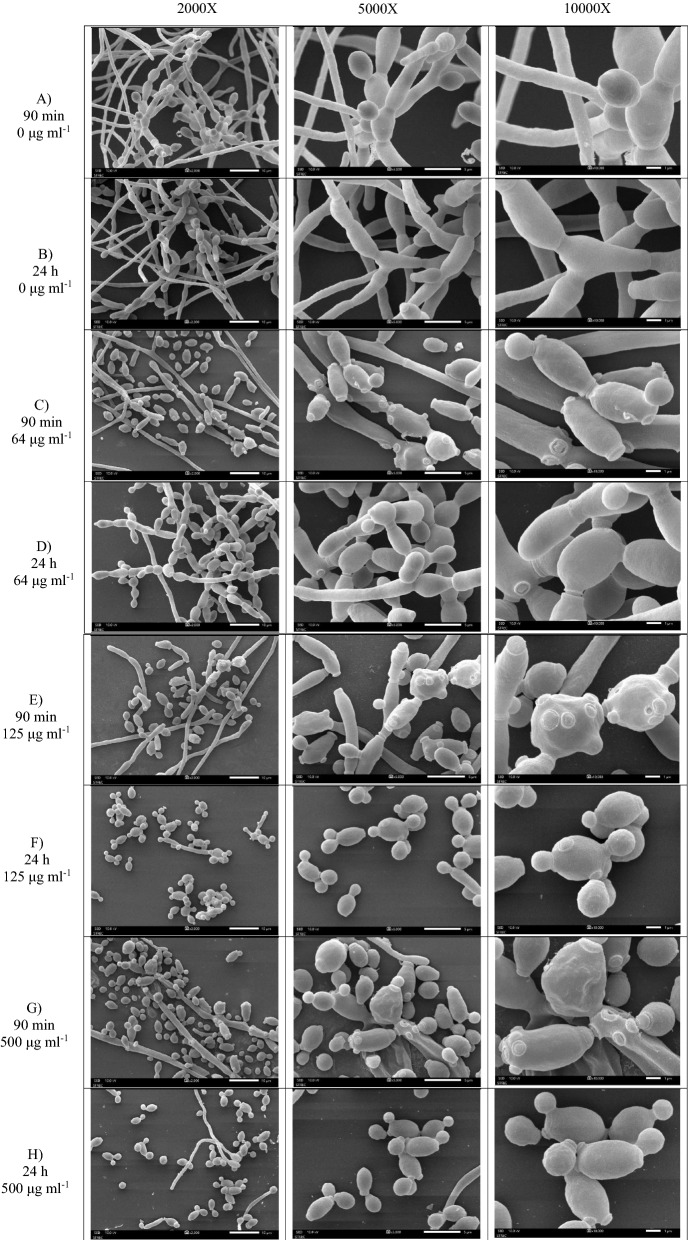
Scanning electron microscopy images of *C. albicans* biofilms. SEM was used to examine the effect of sophorolipids (SLs) on *C. albicans* biofilm formation at different magnification. Biofilms were formed on coated poly-Lysine glass cover slips for 90 min or 24 h at 37 °C. Biofilm formation of *C. albicans* (ATCC 90,028) in the absence (0 µg ml^−1^) of SLs (**A**, **B**), 64 µg ml^−1^of SLs (**C**, **D**), 125 µg ml^−1^ of SLs (**E**, **F**), or  500 µg ml^−1^ of SLs (**G**, **H**)

Biofilm formation and morphological changes are critical for the virulence of a range of plant and human fungal pathogens, including *C. albicans*. The hyphal form contributes to disease by invading epithelial cells and causing tissue damage. The effect of SLs on *C. albicans* biofilm and cellular morphology was visually examined using scanning electron microscopy (SEM). Biofilms of *C. albicans* were formed in the initial stage at 90 min of adhesion step and 24 h of the mature step on glass cover slips. After, different concentrations of purified SLs were applied. In comparison to untreated control sample without SLs, disruption of *C. albicans* biofilm structures and alteration of cell surfaces were found following SLs treatments especially at higher concentration used (Fig. 6). SEM images of untreated control sample (0 μg ml^−1^ of SLs) was observed with tight multilayer of long filamentous yeast cells with intact surfaces and connective septin rings (Fig. 6 A, B). Reduced biofilm was observed following the 90 min and 24 h of SL treatments at concentration of 64 μg ml^−1^, resulting in decreased hyphal formation and increased yeast cells in fragmented forms (Fig. 6C, D). While cell separation is suppressed in hyphae, longer exposure to SLs also appeared to induce septa formation (Fig. 6D). In addition, membrane surface of cells treated with SLs after 90 min or 24 h adhesion showed wrinkled with bud scars that are more evident at higher concentrations of treated SLs (Fig. 6C–H). Growth inhibition of *C. albicans* cells and reduced numbers of hyphae forms (Fig. 6E, F) were shown after treatment with SLs at 125 μg ml^−1^. Cells appeared to be swollen with the presence of multiple bud scars and wrinkled surface (Fig. 6E, F). Finally, at the highest concentration of treated SLs at 500 μg ml^−1^, pronounce effect on defective hyphae formation and biofilm disruption were observed, resulting in lower numbers of remaining yeast cells with swollen, wrinkled, punctured and short fragmented bodies (Fig. 6G, H). Altered cellular morphology shown was consistent with results of XTT reduction assay indicating that SLs decreased the metabolic activity of cells in the adherence phase (90 min) and the mature biofilm (24 h) (Fig. 5A) as well as the reduction of biomass of *C. albicans* biofilm as determined by the crystal violet (CV) assay (Fig. 5B). Overall, SLs are shown to be a potent antimicrobial against *C. albican* as the inhibitor of hyphal and biofilm development.

## Discussion

This study reports the production of high-value biochemical SLs produced by *S. riodocensis* when compared to *S. bombicola*, which is previously discovered as a high potential biosurfactant producer [9]. *S. riodocensis* has been reported by Pimentel et al. [35]; however, less is explored regarding the biological function of SLs produced and their biotechnological applications. Interestingly, we have screened local Thai honey collection for potential biosurfactant yeast producers and identified the GT-SL1R strain closest to the *S. riodocensis.* The oil displacement and emulsification activity of SLs found is consistent with the findings of Youssef et al. [48] that report the clear zone of surfactin biosurfactant. Previously, Gudina et al. [49] have reported that one of the features essentially used for industrial processes is based on the emulsifying activity due to the broad spectrum of utilization which includes distinct hydrophobic counterparts of biosurfactant. For comparison of biosurfactant activity, GT-SL1R *S. riodocensis* was observed to has the emulsification activity of 55% which is similar to *S. bombicola* and higher than those previously reported. Other studies including Camargo et al. [50] reported that *Meyerozyma guilliermondii* and *Rhodotorula glutinis* yeasts performed best emulsion activity at 42.8% and 52.5%, respectively. Subsequently, the *Cryptococcus luteolus*, *Candida orthopsilosis,* and *Hannaela* *sinensis* yeasts showed no emulsion, while *Rhodotorula mucilaginosa*, *Dipodascus australiensis*, *Metschnikowia koreensis* exhibited percentages of emulsification index above 35% with residual soybean oil under an acidic condition. Thus, the result suggested good emulsification ability of produced SLs by GT-SL1R strain using selected substrates (Table 1). Intriguingly, Archana et al. [2] also observed SLs produced by *Candida* sp. AH62 under glucose, oleic acid, yeast extract, and basal media and possessed the maximum emulsion of 76.4%. In general, an emulsifier is considered effective when its emulsification index is more than 40% [26]. In our study, SLs biosurfactant produced by *S. bombicola* recorded better activity to emulsify kerosene (> 60%) as compared to SLs produced by *S. riodocensis* (> 50%) (Table 1). However, our newly *S. riodocensis* GT-SL1R strain can be considered a better yeast producer in term of emulsification activity when compared to those previously studied by Camargo et al. [50]. The use of SLs against kerosene as a long-chain hydrocarbon substrate is the alternative way to improve the biodegradation efficiency in the environment.

Here, shake-flask experiments are carried out and the growth curves are obtained to establish the relationship between cell growth, biosurfactant yield, and the effect of palm oil as a hydrophobic substrate. The yeasts *S. riodocensis* grow better than *S. bombicola* when supplemented with palm oil (Fig. 2). The presence of medium-chain fatty acids composition, such as the decanoic acid content may have a negative effect on the cell viability of *S. bombicola*. Van Bogaert et al. [8] investigated the fatty acid composition and their effect on *C. bombicola* cell viability and showed that decanoic acid (C10:0) or capric acid has a lethal effect on cell viability. Rego et al. [51], on the other hand, explained that lipids serve different functions in the cells as energy and metabolic source, structural elements, signalling molecules, or mediators of apoptosis and membrane fusion. Palm oil supplemented media is found to prolong cell viability and induces the growth of yeast cells [52] particularly in *S. riodocensis*.

Basically, the production of SLs is more high yield when the hydrophilic substrate is supplemented with the hydrophobic substrate in the production media [9]. We use co-utilization of glucose as a hydrophilic substrate and palm oil as a hydrophobic substrate. The use of palm oil is useful to limit the cost of SLs production as compared to other vegetable oils due to the abundantly available plants in Southeast Asia [53, 54]. The SLs yields obtained here are comparable with the values reported in the previous studies. Shah et al. [23] achieved the highest SLs yield of about 32 g l^−1^ using 10% glucose and 10% palm oil compared with tapis oil [26 g l^-1^], melita oil (21 g l^−1^), and ratawi oil (19 g l^−1^). Subsequently, Wadekar et al. [55] reported that the SLs production of *S. bombicola* yeast using sunflower oil and palm oil (10% w/v) as well as supplemented with 15% w/v glycerol as carbon sources were 6.6 g l^−1^ and 5.6 g l^−1^, respectively. Poomtien et al. [53] also reported that palm oil affected crude biosurfactant production by *Cyberlindnera samutprakarnensis* when mixed with glucose. The first report of SLs production using the *S. riodocensis* strain by Kurtzman et al. [6] indicated the production yield of about 8.3 g l^−1^ when cultivated using oleic-containing media. Daverey & Pakshirajan [56] have been reported that yeast requires a limited amount of nitrogen to produce SLs; thus, in this study, the culture media is supplemented with a small amount of urea (1 g l^−1^) to promote SLs production. Montoya et al. [57] reported the composition of palm oil contains approximately 50% saturated fatty acids, such as 44% palmitic acid (C16:0), 5% stearic acid (C18:0), and trace amounts of myristic acid (C14:0). The unsaturated fatty acids are approximately 40% oleic acid (C18:1), 10% polyunsaturated linoleic acid (C18:2), and linolenic acid (C18:3). The higher SLs production by *S. riodocensis* may be due to the effect of vaccenic acid and linoleic acid on SLs production. Although biosurfactants are currently unable to compete economically with chemically surfactant compounds in the markets due to high production costs, different approaches are underway to improve SLs production including metabolic engineering. The underlying genes and enzymes associated with SLs production in *S. bombicola* are recently documented [37]. Alternatively, shake-flask and bioreactor experiments have been reported as common cultivation and scale-up method to produce higher amounts of SLs biosurfactant [8, 23, 44, 56, 58–60].

The compositions of the SLs biosurfactant produced by *S. riodocensis* and *S. bombicola* are determined by TLC, FTIR, and HPLC analyses (Fig. 3). The functional groups present in the crude SLs suggested that crude SLs synthesized by *S. riodocensis* and *S. bombicola* strains were composed of acidic and lactonic forms of SLs (Fig. 3). Similar FTIR spectra are reported in a previous study [44] for SLs production by *Wickerhamiella domercqiae* grown on inorganic compounds, yeast extract, glucose, and oleic acid. Wadekar et al. [55] also confirmed the presence of lactonic and acidic SLs synthesized by *S. bombicola* using 15% glycerol (w/v) and 10% sunflower oil (w/v). The FTIR spectrum of the acidic SLs indicated the ether linkage, carbonyl groups, alkyl chain, and hydroxyl group while the FTIR spectrum of the lactonic SLs showed the presence of ether linkage, carbonyl group, and alkyl chain but no hydroxyl group [55]. The purified SLs synthesized by *S. riodocensis* was then confirmed using standard compounds via HPLC (Fig. 3). According to retention times (RT), the purified SLs contain a mixture of acidic and lactonic forms after comparing with the acidic SL non-acetylated and lactonic di-acetylated standards, consistent with the results confirmed by Kim et al. [25]. In agreement with the previous work by Kurtzman et al. [6], *S. riodocensis* produced structural diversity of SLs, including free acid forms of the monoacetylated and non-acetylated SLs with some lactonic forms.

Regarding the biological activity of SLs, the reduction of hyphal and biofilm formation by SLs is a noteworthy result since hyphae and biofilm formation are crucial virulence factors during *C. albicans* infection [61, 62]. In addition to the involvement in biofilm formation, hyphae facilitate *C. albicans* penetration into host tissues [63]. According to Chen et al. [21] and others’ reports, hyphae are an important part of the disease process and can harm tissue by entering mucosal epithelial cells and causing blood infection. Most mutants that do not produce hyphae are virulently compromised. Here, SLs produced by *S. riodocensis* GT-SL1R strain could inhibit the *Candida* hyphal and biofilm formation and reduce cell viability (Figs. 4 and 5). The biofilm is observed spectrophotometrically with crystal-violet staining and XTT reduction assay to assess biomass biofilm and cell viability, respectively.

SEM of SLs treated biofilms was then examined to observe morphological and structural alterations of *Candida* biofilm. The antimicrobial mechanisms of action of SLs are under studied particularly on antibiofilm and anti-hyphae activity. From SEM results, it is speculated that SLs may inhibit the hyphal growth and development by interfering with normal function of proteins or cellular targets involved in this process as shown by detachment of septum or fragmented septum and the appearance of septin rings and bud scars (Fig. 6C–H). Moreover, cells with wrinkled surface and perforated membrane are observed, following increased SLs concentrations (Fig. 6E–H). SL treatment has been shown to promote leakage of cytoplasmic components in both budding cells and hyphae forms of *C. albicans* in the extracellular matrix of biofilm [11, 12]. The actions of SLs are suggested to be related to the synergistic interactions between the fatty acid and sophorose congeners [64]. Di Pasqua et al. [65] has reported that lipophilic property of antimicrobial compounds enhance the permeability and fluidity of yeast cell membrane, affecting ion transport and balance. Therefore, the hydrophobic part of SLs may contribute to their antimicrobial action against *C. albicans*. Therefore, mechanism of SLs action is warrant future investigation to further improve its antifungal property.

Recently, the development of efficient and eco-friendly antifungal agent SLs and other types of glycolipids with broad applications in food, agriculture, and biomedicines is actively investigated. However, the antimicrobial activity of SLs with reduction of the biofilms and hyphal inhibition against pathogenic microorganisms are rarely reported. Haque et al. [18] reported that 15–30 μg ml^−1^ of SLs can inhibit the metabolic activity of the adherence phase of *C. albicans* by approximately 50%. Subsequently, 120 μg ml^−1^ of SLs can inhibit *C. albicans* mature biofilm by approximately 50%. Although, this study did not well explain the terminology between eradication and inhibition for XTT reduction assay. We report that the purified palm oil-derived SLs produced by *S. riodocensis* can inhibit *C. albicans* 90-min-old biofilm by approximately 50% (125 μg ml^−1^). Higher SLs concentrations are required in this study to inhibit *C. albicans* as compared to those reported by Haque et al. [18]. This may due to different types and composition of SLs produced by yeast species. Using the crystal violet staining, Sen et al. [17] reveal that 250 μg ml^−1^ of SLs can eradicate the biofilm formation of *T. mentagrophytes* while 125 μg ml^−1^ and 500 μg ml^−1^ SLs can reduce *C. albicans* 90-min-old or mature biofilm by approximately 50% in this study, respectively (Fig. 5B). Due to its high antimicrobial potential, many aspects shall be further explored. Previous work has shown that SLs are well-known as low toxicity compounds [1, 9]. Cytotoxicity test is previously conducted and reported by Maeng et al. [66] and Lydon et al. [14] and no cytotoxicity of SLs is observed up to a concentration of 50 µg ml^−1^ in human skin fibroblasts cell culture. Meanwhile, Lydon et al. [14] reported that SL concentrations ranging from 0.01 to 0.5 mg ml^−1^ have no adverse effects on endothelial cells including human umbilical vein and human dermal microvascular and human keratinocyte-derived cell lines. Although low toxicity of SLs is reported previously, future work is required. Optimistically, a study reports the cytotoxicity of SL-and AmB formulation on mature *C. albicans* biofilm and shows that it is lower when compared to an expensive marketed drug phosome, a liposomal formulation of AmB [67]. In addition, since genes associated with hyphal morphology and development are linked to virulence, further examination of a cluster of genes could help to elucidate the involved mechanisms of action of SLs. Thus, SLs are attractive natural biomolecules in biomedicines as adjuvants to existing antifungals against certain infections by inhibiting hyphal growth and/or disrupting biofilms.

## Conclusion

Using palm oil as a hydrophobic substrate, biosurfactant SLs produced by *S. bombicola* and honey-derived GT-2564R *S. riodocensis* display good properties to emulsify kerosene (54.68–60.22%) and displace oil (Table 1). At 48 h., *S. riodocensis* GT-SL1R slightly produces biosurfactant SLs at 40.55 g l^−1^ or a productivity of 0.84 as compared to *S. bombicola* BCC5426 which produces at 39.36 g l^−1^ and productivity of 0.82, respectively (Table 1). Palm oil is an effective low-cost substrate for the production of both lactone and acidic forms of SLs with the yield on the substrate between 31 and 39 or 30–46% using *S. bombicola* or *S. riodocensis* strain, respectively (Table 1). In terms of biological activity, SLs have anti-hyphal and antibiofilm activities against *C. albicans*. The lowest concentration of SLs was found at 125 μg ml^−1^ which can inhibit *C. albicans* in 90-min-old biofilm by approximately 50%. Subsequently, 500 μg ml^−1^ of SLs can inhibit *C. albicans* mature biofilm by approximately 50% within 24 h. Further, the lowest concentration of SLs is found at 125 μg ml^−1^ which can reduce the biofilm biomass by approximately 50% as compared to control. Importantly, this study showed that SLs produced by *S. riodocensis* could reduce hyphal growth of key pathogenic yeast *C. albicans* as shown by SEM analysis although the mechanism of action remains to be elucidated. Moreover, characterization of SLs biosynthetic genes in *S. riodocensis* will be beneficial in terms of the production. To this end, yeasts including *S. riodocensis* have high potential as a microbial cell factory for the production of environmental-friendly biosurfactants, serving the increasing demand for future uses in functional food, agriculture, and industries as well as health and well-being.

## References

[1] Sen S, Borah SN, Bora A, Deka S (2017). Production, characterization, and antifungal activity of a biosurfactant produced by *Rhodotorula babjevae* YS3. Microb Cell Fact.

[2] Archana K, Sathi Reddy K, Parameshwar J, Bee H (2019). Isolation and characterization of sophorolipid producing yeast from fruit waste for application as antibacterial agent. Environ Sustain.

[3] Santos DK, Rufino RD, Luna JM, Santos VA, Sarubbo LA (2016). Biosurfactants: multifunctional biomolecules of the 21st century. Int J Mol Sci.

[4] Yan X, Gu S, Cui X, Shi Y, Wen S, Chen H (2019). Antimicrobial, anti-adhesive and anti-biofilm potential of biosurfactants isolated from *Pediococcus acidilactici* and *Lactobacillus plantarum* against *Staphylococcus aureus* CMCC26003. Microb Pathog.

[5] Liu J, Li W, Zhu X, Zhao H, Lu Y, Zhang C (2019). Surfactin effectively inhibits *Staphylococcus aureus* adhesion and biofilm formation on surfaces. Appl Microbiol Biotechnol.

[6] Kurtzman CP, Price NP, Ray KJ, Kuo TM (2010). Production of sophorolipid biosurfactants by multiple species of the *Starmerella* (*Candida*) *bombicola* yeast clade. FEMS Microbiol Lett.

[7] Konishi M, Morita T, Fukuoka T, Imura T, Uemura S, Iwabuchi H (2017). Selective production of acid-form sophorolipids from glycerol by *Candida floricola*. J Oleo Sci.

[8] Van Bogaert INA, Roelants S, Develter D, Soetaert W (2010). Sophorolipid production by *Candida bombicola* on oils with a special fatty acid composition and their consequences on cell viability. Biotech Lett.

[9] Van Bogaert INA, Zhang J, Soetaert W (2011). Microbial synthesis of sophorolipids. Process Biochem.

[10] Kulakovskaya EKT (2013). Extracellular glycolipids of yeasts: biodiversity, biochemistry, and prospects.

[11] de Caretta OT, Silveria VAI, Andrade G, Macedo F, Celligoi PCMA (2022). Antimicrobial activity of sophorolipids produced by *Starmerella bombicola* against phytopathogens from cherry tomato. J Sci Food Agric.

[12] Park I, Oh S, Nam H, Celi P, Lillehoj HS (2022). Antimicrobial activity of sophorolipids against *Eimeria maxima* and *Clostridium perfringens*, and their effect on growth performance and gut health in necrotic enteritis. Poult Sci.

[13] Ceresa C, Fracchia L, Williams M, Banat IM, De DiazRienzo MA (2020). The effect of sophorolipids against microbial biofilms on medical-grade silicone. J Biotechnol.

[14] Lydon HL, Baccile N, Callaghan B, Marchant R, Mitchell CA, Banat IM (2017). Adjuvant antibiotic activity of acidic sophorolipids with potential for facilitating wound healing. Antimicrob Agents Chemother.

[15] Kim K, Dalsoo Y, Youngbum K, Baekseok L, Doonhoon S (2002). Characteristics of sophorolipid as an antimicrobial agent. J Microbiol Biotechnol..

[16] Tang Y, Ma Q, Du Y, Ren L, Van Zyl LJ, Long X (2020). Efficient purification of sophorolipids via chemical modifications coupled with extractions and their potential applications as antibacterial agents. Sep Purif Technol.

[17] Sen S, Borah SN, Kandimalla R, Bora A, Deka S (2020). Sophorolipid biosurfactant can control cutaneous dermatophytosis caused by *Trichophyton mentagrophytes*. Front Microbiol.

[18] Haque F, Alfatah M, Ganesan K, Bhattacharyya MS (2016). Inhibitory effect of sophorolipid on *Candida albicans* biofilm formation and hyphal growth. Sci Rep.

[19] Theberge S, Semlali A, Alamri A, Leung KP, Rouabhia MC (2013). Albicans growth, transition, biofilm formation, and gene expression modulation by antimicrobial decapeptide KSL-W. BMC Microbiol.

[20] Pappas PG, Lionakis MS, Arendrup MC, Ostrosky-Zeichner L, Kullberg BJ (2018). Invasive candidiasis. Nat Rev Dis Prim.

[21] Chen H, Zhou X, Ren B, Cheng L (2020). The regulation of hyphae growth in *Candida albicans*. Virulence.

[22] Kumari A, Kumari S, Prasad GS, Pinnaka AK (2021). Production of Sophorolipid biosurfactant by insect derived novel yeast *Metschnikowia churdharensis* f.a, sp. nov., and its antifungal activity against plant and human pathogens. Front Microbiol.

[23] Shah MUH, Sivapragasam M, Moniruzzaman M, Talukder MMR, Yusup SB, Goto M (2017). Production of sophorolipids by *Starmerella bombicola* yeast using new hydrophobic substrates. Biochem Eng J.

[24] Daverey A, Pakshirajan K (2009). Production of sophorolipids by the yeast *Candida bombicola* using simple and low cost fermentative media. Food Res Int.

[25] Kim JH, Oh YR, Hwang J, Kang J, Kim H, Jang YA (2021). Valorization of waste-cooking oil into sophorolipids and application of their methyl hydroxyl branched fatty acid derivatives to produce engineering bioplastics. Waste Manag.

[26] Araujo S, Silva-Portela RCB, de Lima DC, da Fonseca MMB, Araujo WJ, da Silva UB (2020). MBSP1: a biosurfactant protein derived from a metagenomic library with activity in oil degradation. Sci Rep.

[27] Aguiar FLL, Santos NC, de Paula Cavalcante CS, Andreu D, Baptista GR, Goncalves S (2020). Antibiofilm activity on *Candida albicans* and mechanism of action on biomembrane models of the antimicrobial peptide ctn. Int J Mol Sci.

[28] Di Giulio M, Zappacosta R, Di Lodovico S, Di Campli E, Siani G, Fontana A (2018). Antimicrobial and antibiofilm efficacy of graphene oxide against chronic wound microorganisms. Antimicrob Agents Chemother.

[29] Kuhn DM, Balkis M, Chandra J, Mukherjee PK, Ghannoum MA (2003). Uses and limitations of the XTT assay in studies of *Candida* growth and metabolism. J Clin Microbiol.

[30] Khan SN, Khan S, Iqbal J, Khan R, Khan AU (2017). Enhanced killing and antibiofilm activity of encapsulated cinnamaldehyde against *Candida albicans*. Front Microbiol.

[31] Dong J, Signo KSL, Vanderlinde EM, Yost CK, Dahms TES (2011). Atomic force microscopy of a ctpA mutant in *Rhizobium leguminosarum* reveals surface defects linking CtpA function to biofilm formation. Microbiology.

[32] Can Z, Yildiz O, Sahin H, Turumtay EA, Silici S, Kolayli S (2015). An investigation of Turkish honeys: their physico-chemical properties, antioxidant capacities and phenolic profiles. Food Chem.

[33] Zahoor F, Sooklim C, Songdech P, Duangpakdee O, Soontorngun N (2021). Selection of potential yeast probiotics and a cell factory for xylitol or acid production from honeybee samples. Metabolites.

[34] de Oliveria Scoaris D, Hughes FM, Silveira MA, Evans JD, Pettis JS, Bastos EM (2021). Microbial communities associated with honey bees in Brazil and in the United States. Braz J Microbiol.

[35] Pimentel MR, Antonini Y, Martins RP, Lachance MA, Rosa CA (2005). *Candida riodocensis* and *Candida cellae*, two new yeast species from the *Starmerella* clade associated with solitary bees in the Atlantic rain forest of Brazil. FEMS Yeast Res.

[36] Kurtzman CP, Robnett CJ (1997). Identification of clinically important ascomycetous yeasts based on nucleotide divergence in the 5' end of the large-subunit (26S) ribosomal DNA gene. J Clin Microbiol.

[37] Qazi MA, Wang Q, Dai Z (2022). Sophorolipids bioproduction in the yeast *Starmerella bombicola*: current trends and perspectives. Biores Technol.

[38] Lachance MA, Dobson J, Wijayanayaka DN, Smith AM (2010). The use of parsimony network analysis for the formal delineation of phylogenetic species of yeasts: *Candida apicola, Candida azyma*, and *Candida parazyma* sp. nov., cosmopolitan yeasts associated with floricolous insects. Antonie Van Leeuwenhoek.

[39] Vu D, Groenewald M, Szöke S, Cardinali G, Eberhardt U, Stielow B (2016). DNA barcoding analysis of more than 9000 yeast isolates contributes to quantitative thresholds for yeast species and genera delimitation. Stud Mycol.

[40] Fell JW, Boekhout T, Fonseca A, Scorzetti G, Statzell-Tallman A (2000). Biodiversity and systematics of basidiomycetous yeasts as determined by large-subunit rDNA D1/D2 domain sequence analysis. Int J Syst Evol Microbiol.

[41] Schoch CL, Robbertse B, Robert V, Vu D, Cardinali G, Irinyi L (2014). Finding needles in haystacks: linking scientific names, reference specimens and molecular data for Fungi. Database.

[42] Bahia M, Passos F, Adarme OF, Aquino SF, Silva SQ (2018). Anaerobic-aerobic combined system for the biological treatment of azo dye solution using residual yeast: Bahia et al. Water Environ Res.

[43] Asmer H-J, Lang S, Wagner F, Wray V (1988). Microbial production, structure elucidation and bioconversion of sophorose lipids. J Am Oil Chem Soc.

[44] Ma XJ, Li H, Shao LJ, Shen J, Song X (2011). Effects of nitrogen sources on production and composition of sophorolipids by *Wickerhamiella domercqiae* var. sophorolipid CGMCC 1576. Appl Microbiol Biotechnol.

[45] Yalcin HT, Ergin-Tepebasi G, Uyar E (2018). Isolation and molecular characterization of biosurfactant producing yeasts from the soil samples contaminated with petroleum derivatives. J Basic Microbiol.

[46] Uppuluri P, Acosta Zaldivar M, Anderson MZ, Dunn MJ, Berman J, Lopez Ribot JL (2018). *Candida albicans* dispersed cells are developmentally distinct from biofilm and planktonic cells. mBio.

[47] Corte L, Casagrande Pierantoni D, Tascini C, Roscini L, Cardinali G (2019). Biofilm specific activity: a measure to quantify microbial biofilm. Microorganisms.

[48] Youssef NH, Duncan KE, Nagle DP, Savage KN, Knapp RM, McInerney MJ (2004). Comparison of methods to detect biosurfactant production by diverse microorganisms. J Microbiol Method.

[49] Gudina EJ, Pereira JF, Costa R, Evtuguin DV, Coutinho JA, Teixeira JA (2015). Novel bioemulsifier produced by a *Paenibacillus* strain isolated from crude oil. Microb Cell Fact.

[50] Camargo FP, Menezes AJ, Tonello PS, Dos Santos ACA, Duarte ICS (2018). Characterization of biosurfactant from yeast using residual soybean oil under acidic conditions and their use in metal removal processes. FEMS Microbiol Lett.

[51] Rego A, Trindade D, Chaves SR, Manon S, Costa V, Sousa MJ (2014). The yeast model system as a tool towards the understanding of apoptosis regulation by sphingolipids. FEMS Yeast Res.

[52] Escriba PV, Gonzalez-Ros JM, Goni FM, Kinnunen PK, Vigh L, Sanchez-Magraner L (2008). Membranes: a meeting point for lipids, proteins and therapies. J Cell Mol Med.

[53] Poomtien J, Thaniyavarn J, Pinphanichakarn P, Jindamorakot S, Morikawa M (2013). Production and characterization of a biosurfactant from *Cyberlindnera samutprakarnensis* JP52(T). Biosci Biotechnol Biochem.

[54] Yusoff MNAM, Zulkifli NWM, Sukiman NL, Chyuan OH, Hassan MH, Hasnul MH (2021). Sustainability of palm biodiesel in transportation: a review on biofuel standard, policy and international collaboration between Malaysia and Colombia. BioEnerg Res.

[55] Wadekar SD, Kale SB, Lali AM, Bhowmick DN, Pratap AP (2012). Utilization of sweetwater as a cost-effective carbon source for sophorolipids production by *Starmerella bombicola* (ATCC 22214). Prep Biochem Biotechnol.

[56] Daverey A, Pakshirajan K (2010). Sophorolipids from *Candida bombicola* using mixed hydrophilic substrates: production, purification and characterization. Coll Surf B Biointerfaces.

[57] Montoya C, Cochard B, Flori A, Cros D, Lopes R, Cuellar T (2014). Genetic architecture of palm oil fatty acid composition in cultivated oil palm (*Elaeis guineensis* Jacq.) compared to its wild relative *E. oleifera* (H.B.K) Cortes. PLoS ONE.

[58] Fleurackers SJ, Van Bogaert IN, Develter D (2010). On the production and identification of medium-chained sophorolipids. Eur J Lipid Sci Technol.

[59] Roelants SL, Ciesielska K, De Maeseneire SL, Moens H, Everaert B, Verweire S (2016). Towards the industrialization of new biosurfactants: Biotechnological opportunities for the lactone esterase gene from *Starmerella bombicola*. Biotechnol Bioeng.

[60] De Graeve M, Van de Velde I, Saey L, Chys M, Oorts H, Kahriman H (2019). Production of long-chain hydroxy fatty acids by *Starmerella bombicola*. FEMS Yeast Res.

[61] Kumamoto CA, Vinces MD (2005). Contributions of hyphae and hypha-co-regulated genes to *Candida albicans* virulence. Cell Microbiol.

[62] Gulati M, Nobile CJ (2016). Candida albicans biofilms: development, regulation, and molecular mechanisms. Microbes Infect.

[63] Sudbery PE (2011). Growth of *Candida albicans* hyphae. Nat Rev Microbiol.

[64] Silveira VAI, Nishio EK, Freitas CAUQ, Amador IR, Kobayashi RKT, Caretta T (2019). Production and antimicrobial activity of sophorolipid against *Clostridium perfringens* and *Campylobacter jejuni* and their additive interaction with lactic acid. Biocatal Agric Biotechnol.

[65] Di Pasqua R, Betts G, Hoskins N, Edwards M, Ercolini D, Mauriello G (2007). Membrane toxicity of antimicrobial compounds from essential oils. J Agric Food Chem.

[66] Maeng Y, Kim KT, Zhou X, Jin L, Kim KS, Kim YH (2018). A novel microbial technique for producing high-quality sophorolipids from horse oil suitable for cosmetic applications. Microb Biotechnol.

[67] Haque F, Sajid M, Cameotra SS, Battacharyya MS (2017). Anti-biofilm activity of a sophorolipid-amphotericin B niosomal formulation against *Candida albicans*. Biofouling.

